# Racial and ethnic disparities in Alzheimer’s dementia

**DOI:** 10.18632/aging.204421

**Published:** 2022-11-29

**Authors:** Unhee Lim, Song-Yi Park

**Affiliations:** 1 Population Sciences in the Pacific Program, Cancer Center, University of Hawaii at Manoa, Honolulu, HI, USA

**Keywords:** Alzheimer’s disease and related dementias, racial and ethnic disparities, population-based cohort studies

With the aging of the US population, late-onset Alzheimer’s disease and related dementias (ADRD) are projected to double in prevalence and reach 12.7 million cases by year 2050 [1]. In particular, ADRD incidence has been reported to differ by race and ethnicity, with higher rates observed among African Americans and Caribbean Latinos in studies that compared one or two minority groups to non-Hispanic White individuals [2]. However, population-based cohort data have been sparse to directly compare the risk of ADRD across multiple racial and ethnic populations. In analysis of a large health care delivery system in Northern California, age-adjusted dementia incidence, based on the first occurrence of diagnosis in the medical records, varied 1.7-fold by race and ethnicity, highest in African American group, followed by American Indian and Alaska Native, Mexican American, a small number of Pacific Islander, White and Asian American groups [3]. The study underscored the substantial, however, lacked information on some key risk factors, such as education, history of cardiometabolic conditions in midlife, lifestyle exposures, and apolipoprotein E (*APOE*) genotype, which limited the understanding of the disparities.

In this context, we recently examined AD and ADRD incidence rates in the Multiethnic Cohort (MEC; 1993-present), established from a large population sample of middle-aged and older adult residents of Hawaii and Los Angeles County, California [4]. Based on the MEC linkage with the Medicare claims for fee-for-service beneficiaries, and applying careful strategies to capture diagnostic incidence, we identified ~7,400 AD and ~16,400 ADRD cases among 105,800 MEC participants over up to 14 years of follow-up. The resulting age-adjusted diagnostic incidence rates of AD and ADRD showed a nearly 2-fold difference across six racial and ethnic groups in the MEC, showing the highest dementia risk among African American individuals and the lowest risk among Asian American individuals of Japanese or Filipino descent. Also, we found the expected evidence that some of the established risk factors (education, midlife history of cardiometabolic conditions) likely mediated part of the racial and ethnic disparities in AD/ADRD risks. These findings were consistent with prior studies based on clinical assessment despite the limitations in our data, where diagnosis codes in the Medicare claims were used for AD/ADRD definitions.

There were some unique strengths to our analysis and findings that provide further insights. Considering that AD/ADRD are highly dependent on age and aging-associated comorbidities, we additionally performed a Fine-Gray competing risk model. This attenuated the racial and ethnic differences in AD/ADRD risks, indicating that the 2-fold difference in our main results would have been larger were it not for more frequent premature deaths from other competing diseases in higher-risk racial and ethnic groups. Ours was the first study of Native Hawaiian group (*n* > 7,000), disaggregated from Asian American and other heterogeneous Pacific Islander groups. We observed a significantly higher ADRD rate in Native Hawaiian compared to White group (19.7 vs. 16.3 per 1,000 person-years) and a high risk for AD among Native Hawaiian men, even above the risk seen among African American men.

In a subset of the MEC participants with known *APOE* genotype, we observed approximate doubling of the risk of AD and ADRD with each additional copy of the ε4 risk allele. A higher proportion of ε4 risk allele carriers was detected among African American individuals (37%), as documented [5], and also Native Hawaiian individuals (35%) compared with others (20%–23%), suggesting that these two groups’ elevated risks in part stem from greater genetic susceptibility. We found evidence of larger racial and ethnic AD/ADRD disparities among non-carriers of *APOE* ε4 compared to carriers. On the other hand, accounting for the unequal distribution of modifiable risk factors and *APOE* ε4 risk allele only slightly reduced the extent of ADRD disparities, signifying the contribution of other yet-undescribed risk factors in our data, such as hearing loss, history of depression, air pollution, and social determinants of health [6, 7].

As the proportion of racial and ethnic minorities is growing in the overall US population and among older adults [8], it is paramount to improve the minority representation in biomedical research, especially of ADRD that present with large variation in risk by race and ethnicity. Leveraging existing population-based cohorts that were not specifically designed for AD/ADRD research, like the MEC, provides an opportunity to better understand ADRD etiology around varying distribution of risk factors across groups with both higher and lower risk profiles compared to non-Hispanic White population. Ultimately, race- and ethnicity-specific data and knowledge base will enable the development of optimal precision medicine approaches for the prevention and treatment of ADRD.
